# Characteristics of cases and deaths arising from SARS-CoV-2 infection in Zambia: March 2020 to April 2021

**DOI:** 10.11604/pamj.2023.45.155.32018

**Published:** 2023-08-10

**Authors:** Stephen Longa Chanda, Emmanuel Tembo, Nyambe Sinyange, Nkomba Kayeyi, Kunda Musonda, Orbie Chewe, Mpanga Kasonde, Otridah Kapona, Albertina Ngomah, Amos Hamukale, Paul Msanzya Zulu, Muzala Kapina

**Affiliations:** 1 Field Epidemiology Training Program (FETP), Zambia National Public Health Institute, Lusaka, Zambia; 2 Zambia National Public Health Program, Surveillance and Disease Intelligence Cluster, Lusaka, Zambia

**Keywords:** COVID-19, mortality, SARS-CoV-2, demography, Zambia, Africa

## Abstract

**Introduction:**

since March 2020, Zambia has been experiencing a SARS-CoV-2 epidemic. Little data has been reported on cases and deaths arising from COVID-19 in Africa. We described the demographic characteristics of these cases and deaths in Zambia.

**Methods:**

we analyzed data on all persons testing positive for SARS-CoV-2 from 18^th^ March 2020 to 25^th^ April 2021 in Zambia. COVID-19 cases were identified through port-of-entry surveillance, contact-tracing, health-care-worker testing, health-facility-based and community-based screening and community-death screening. All diagnoses were confirmed using real-time-polymerase-chain-reaction and rapid-antigen-test-kits of nasopharyngeal specimens. We analyzed age, sex, and date-of-reporting according to whether the cases or deaths occurred during the first wave (1^st^ July to 15^th^ September 2020) or the second wave (15^th^ December 2020 to 10^th^ April 2021). We computed Mann-Whitney-U-test to compare medians of continuous variables and chi-square tests to compare differences between proportions using R.

**Results:**

a total 1,246 (1.36%) deaths were recorded among 91,378 confirmed cases during March 2020-April 2021 in Zambia. Persons who died were older than those who did not (median age 50 years versus 32.0 years, p< 0.001). Although only 4.7% of cases were among persons aged >60 years, most deaths (31.6%) occurred in this age group (p<0.001). More deaths (83.5%) occurred in the community than in health facilities (p<0.001).

**Conclusion:**

during the SARS-CoV-2 epidemic in Zambia, most deaths occurred in the community, indicating potential gaps in public health messaging about COVID-19. Improving health-seeking behaviors for COVID-19 through public messaging campaigns and engaging key community stakeholders in Zambia might reduce avoidable mortality. As the group most impacted by COVID-19 mortality, older persons might need enhanced outreach and linkage to care.

## Introduction

COVID-19 is an infectious viral disease caused by a novel enveloped RNA beta-coronavirus named severe acute respiratory syndrome coronavirus 2 (SARS-CoV-2) [1]. Most cases are asymptomatic, few present with mild symptoms, and a minority progress to acute respiratory illness and hypoxia requiring hospitalization, and a subset develop acute respiratory distress syndrome, multi-organ failure, or have fatal outcomes [2]. From 18^th^ March 2020 to April 25^th^, 2021, Zambia recorded over 91,000 confirmed cases with over 1000 deaths [3]. Zambia reported its first wave from July to August 2020 and with it an increase in the number of recorded deaths. After several months of low confirmed case counts and deaths, a second wave began in December 2020 and with it an increase in the number of COVID-19-associated deaths [3]. Socio-demographic differences have been reported between people infected in the first and second waves, with the first wave affecting an older segment of the population than the second [4,5]. Age and sex have been shown to affect both the severity and mortality associated with COVID-19, with male sex and the elderly at greater risk of both severe disease and death [2]. A post-mortem surveillance project of COVID-19 in Zambia showed that most SARS-CoV-2 positive deaths occurred in the community and were associated with older age and male sex [6]. However, this study was limited to only one large hospital in Lusaka and the data analyzed was for only four months duration. Another study at this hospital showed that community deaths from all causes also increased in Lusaka during these two COVID-19 peaks [7]. Description of the socio-demographic characteristics of people infected and of those who died from COVID-19 at various stages during the pandemic will help identify high-risk groups that should be targeted for public health interventions towards outbreak control. We describe the demographic characteristics of all the confirmed SARS-CoV-2 positive cases and deaths in Zambia between 18^th^ March 2020 and April 25^th^, 2021.

## Methods

**Study design:** this is a retrospective analysis of the Zambia COVID-19 database currently held at the Zambia National Public Health Institute (ZNPHI). This database contains the names, sex, age, location, clinical outcome, and places of death (health facilities or community) of all reported SARS-CoV-2 positive cases in Zambia from the date of outbreak inception in Zambia (18^th^ March 2020). It is currently held by ZNPHI and is updated on a daily basis with test results from all health facilities and laboratories conducting SARS-CoV-2 testing in Zambia. We chose this database for our investigation as it is the most complete and up-to-date database containing details on all SARS-CoV-2 positive cases in Zambia. By using this study design, we were able to generate evidence about COVID-19 cases and mortality in Zambia using routine programmatic data that can guide the COVID-19 public health response in Zambia, and other similar African countries.

**Study setting:** persons with suspected COVID-19 in Zambia were identified through various mechanisms, including port-of-entry surveillance, contact tracing, health care worker testing, health facility-based screening, mortality surveillance, community-based screenings, and calls from the public into a COVID-19 national hotline administered by the Disaster Management and Mitigation Unit and ZNPHI. All COVID-19 diagnoses were confirmed using real-time-reverse-transcription-polymerase-chain-reaction (RT-PCR) or rapid-diagnostic-testing kits of nasopharyngeal specimens [8]. We analyzed data from the outbreak inception to the end of the second wave in Zambia (April 25^th^, 2021).

**Study population:** a COVID-19 case was defined as a positive SARS-CoV-2 DNA polymerase chain reaction (PCR) or positive SARS-CoV-2 rapid diagnostic test (RDT). A COVID-19 death was defined as a death including people who have had a positive PCR or RDT test for COVID-19, who died without fully recovering from COVID-19 or a positive PCR or RDT after death. A COVID-19 community death was defined as a death that occurs outside of a hospital or healthcare facility or within 24 hours of admission at a health facility with a history of a positive SARS-CoV-2 test prior to death or shortly after death. In Zambia, deaths occurring within the community are required by law to be registered before access to a burial site. This registration takes place at a health facility mortuary before burial. During the COVID-19 pandemic, community deaths were tested for SARS-CoV-2 before being deposited in the mortuary. We defined a hospital/facility SARS-CoV-2 death as a death that occurs after more than 24 hours of admission to a health facility or a hospital with a positive SARS-CoV-2 test. We identified all laboratory-confirmed SARS-CoV-2 positive cases among persons in Zambia. To be eligible, positive cases had to fall within the study period.

**Data extraction:** the following variables were extracted from the database; age, sex, date of result reporting (by the laboratory), clinical outcome, and place of death (health facility or community). There were inconsistencies in the completeness of these variables within the dataset.

**Data analysis:** we stratified the continuous variable age into a categorical variable age group for ease of interpretation. The case fatality proportion was defined as the number of deaths among persons with confirmed cases divided by the number of persons with confirmed cases. The first and second waves were identified by visually inspecting the epidemic curve and noting deflections from the baseline. We analyzed the data according to whether SARS-CoV-2 infections or deaths occurred during the first wave (July 1^st^ to September 15^th^, 2020) or the second wave (December 15^th^, 2020, to April 25^th^, 2021). Additionally, we analyzed the sex-age distribution of all positive cases by wave, we also analyzed the sex-age distribution of deaths by place of death. We compared case fatality proportion by age, sex and wave. Data analysis was done in R version 4.0.4. Categorical variables were summarized as proportions. Simple descriptive statistics were performed. Shapiro-Wilks´s test was used to test for the normality of the distribution of the age variable. Mann-Whitney U test was used to compare the median age of COVID-19 cases and COVID-19 deaths and to compare the median age of all cases and deaths during the first and second waves. The Chi-square test was used to test for the significance of differences between the proportion of all SARs-CoV-2 cases and deaths by age group, clinical outcomes (dead or alive), place of death (facility or community), and sex between the first wave and second wave. Additionally, we used the Chi-square test to test the significance of differences between proportions of age groups, wave periods, sex, and place of death by clinical outcome. A p-value of less than 0.05 was used to make an inference of a statistically significant association.

**Ethical consideration:** ethical approval was obtained to conduct this study from ERES Converge [IRB: 00005948, FWA: 00011697], reference number 2021-JUN-016.

## Results

**Figure 1:**
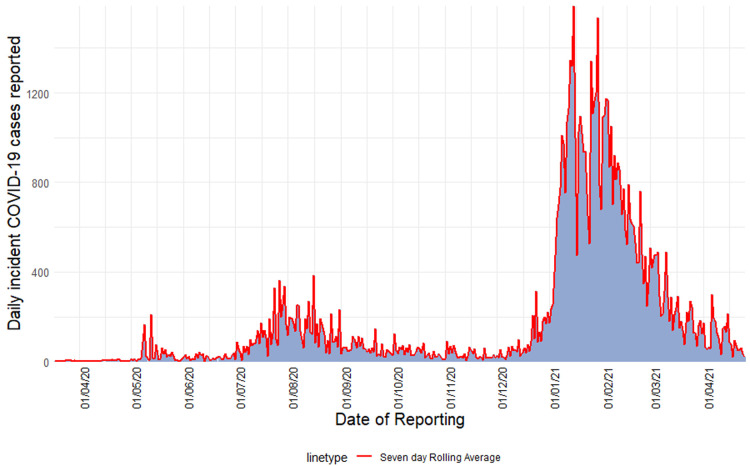
epidemic curve of seven-day rolling average of incident COVID-19 positive cases in Zambia; March 2020-April 2021

**Figure 2:**
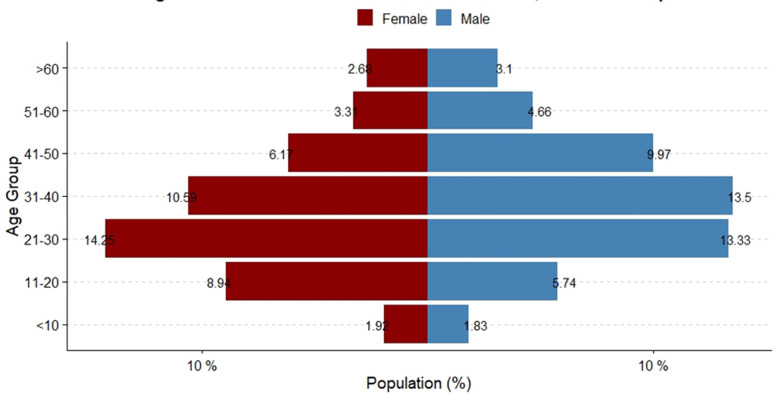
sex and age distribution of COVID-19 cases in Zambia; March 2020-April 2021

**Characteristics of cases:** from March 18^th^, 2020, to April 25^th^, 2021, a total of 91 378 SARS-CoV-2 cases were recorded in Zambia over two waves (Figure 1). The median age amongst cases was 32.0 years (interquartile range [IQR] = 20.0) (Table 1). Most cases (27.4%) were recorded among persons aged 21-30 (p<0.001) (Figure 2). More males tested positive for SARS-CoV-2 (52.3%, p=0.008). More SARS-CoV-2 cases were recorded in the second wave (85.6%, p<0.001). Cases in the first wave were older (median age 37.0 years [IQR = 19] versus 31.0 years [IQR = 20]; p<0.001) (Table 2). The proportion of females affected in the first wave was less than that affected in the second wave (39.3% first wave vs 49.3% second wave, p<0.001).

**Table 1:** comparison of demographic characteristics of SARS-CoV-2 positive people by outcome in Zambia March 2020 - January 2021

		Outcome		
		Alive n = 90555	Dead n = 823	P value
**Age**	Median (IQR)	32.0 (20.0)	50.0 (35.8)	<0.001
**Age group, n (%)**	<10	2775 (3.1)	41 (5.0)	<0.001
	11-20	10852 (12.0)	32 (3.9)	
	21-30	20394 (22.5)	72 (8.7)	
	31-40	17816 (19.7)	116 (14.1)	
	41-50	11930 (13.2)	110 (13.4)	
	51-60	5893 (6.5)	77 (9.4)	
	>60	4273 (4.7)	260 (31.6)	
	(Missing)	16622 (18.4)	115 (14.0)	
**Wave, n (%)**	First wave	11967 (13.2)	295 (35.8)	<0.001
	Second wave	71186 (78.6)	432 (52.5)	
	(Missing)	7402 (8.2)	96 (11.7)	
**Sex, n (%)**	Male	45162 (49.9)	469 (57.0)	0.008
	Female	41266 (45.6)	354 (43.0)	
	(Missing)	4127 (4.6)	0 (0.0)	
**Place of death, n (%)**	Community	0 (0.0)	687 (83.5)	1.000
	Health facility	0 (0.0)	136 (16.5)	

**Table 2:** comparison of demographic characteristics and outcomes of SARS-CoV-2 positive cases between the first and second waves of COVID-19 in Zambia; March 2020 - April 2021

		Wave		
		First n = 12262	Second n = 71618	P -value
**Proportion of total cases %**		13.4	78.4	
**Age**	Median (IQR)	37.0 (19.0)	31.0 (20.0)	<0.001
**Age group, n (%)**	<10	320 (3.4)	2207 (3.7)	<0.001
	11 -20	503 (5.4)	9585 (16.3)	
	21-30	2109 (22.6)	16627 (28.2)	
	31-40	2609 (28.0)	13758 (23.3)	
	41-50	2052 (22.0)	8880 (15.1)	
	51-60	957 (10.3)	4480 (7.6)	
	>60	779 (8.4)	3400 (5.8)	
**Outcome, n (%)**	Alive	11967 (97.6)	71186 (99.4)	<0.001
	Dead	295 (2.4)	432 (0.6)	
**Place of death, n (%)**	Community	218 (73.9)	415 (96.1)	<0.001
	Health Facility	77 (26.1)	17 (3.9)	
**Sex, n (%)**	Male	7392 (60.7)	34382 (50.7)	<0.001
	Female	4794 (39.3)	33454 (49.3)	

**Figure 3:**
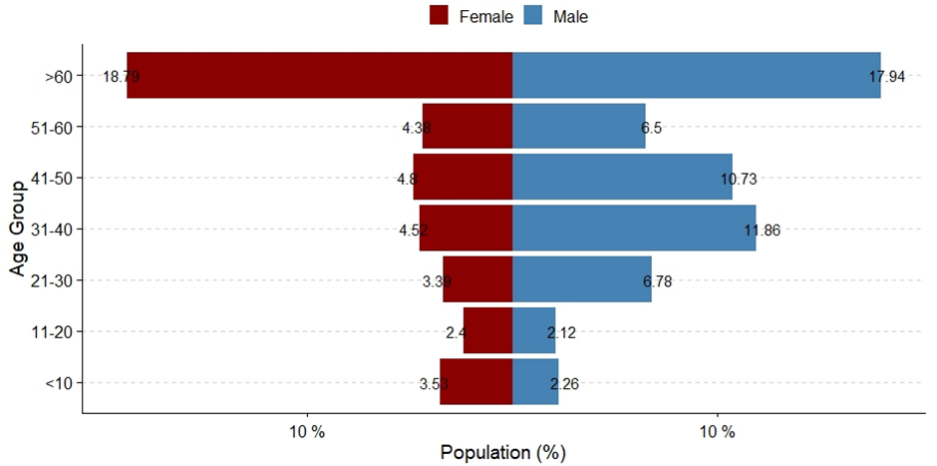
sex and age distribution of COVID-19 deaths in Zambia; March 2020-January 2021

**Characteristics of deaths:** a total of 1,246 (1.36%) deaths were reported among the 91,378 confirmed cases during March 2020-April 2021 in Zambia. The data set contained information on 823 (66.1%) of these deaths. Compared to cases, deaths were older (median age 32.0 years [IQR=20.0] among cases versus median age 50 [IQR=35.8] amongst deaths; p<0.001) (Table 1). Although only 4.7% of cases were among persons aged >60 years, most deaths (31.6%) occurred in this age group (p<0.001) (Figure 3). There were more recorded deaths among males (57.0%, p= 0.008). More deaths occurred in the second wave (52.5% vs 35.8%, p<0.001). More deaths occurred in the community than in health facilities (83.5%, p<0.001). From the data analyzed, from March 18^th^, 2020, to April 25^th^, 2021, the overall case fatality proportion due to COVID-19 was 0.9% in Zambia. The first wave had a higher case fatality (2.4% versus 0.6%, p<0.001). Case fatality was higher among males than females (1.0% versus 0.9%, p< 0.001).

## Discussion

Over two-thirds of all COVID-19 deaths occurred in the community. This could be because of barriers to accessing health services by most of the population with severe disease. This observation is not unique to this study, according to the Zambia sample vital registration with verbal autopsy report, 40.4% of all recorded deaths occurred in the community, with only 53.1% occurring in health facilities [9]. Community deaths from all causes spiked in Lusaka during COVID-19 waves in 2020, suggesting a likely increased number of deaths related to COVID-19 than official statistics capture [6,7]. Therefore, routine surveillance of these community deaths should form part of nationwide COVID-19 surveillance. Further research is required to identify barriers to access to health care in Zambia. In addition, public health messaging strategies are needed to improve health-seeking behavior for COVID-19 in Zambia. The median age of reported SARS-COV-2 infections over the entire outbreak in Zambia was lower than what has been observed worldwide and within the region [1,4,5,10]. This could be due to the younger demographic age structure in Zambia [9]. There was a decline in the median age of cases from the first wave to the second. A similar decline in age between the first and second waves has been reported in both the United States and Europe [4,5]. Changes in testing strategies across age groups could affect the distribution of positive results, or this change could reflect a demographic transition in the segment of the population affected. Further investigations are required to understand this observation as young people make up a large proportion of the Zambian population [9], and may be at higher risk of exposure to SARS-COV-2 because of behavioral and occupational factors.

A larger proportion of cases were male, proportions of reported COVID-19 cases have been shown to vary by sex [11]. Globally, the proportion of male-to-female cases is almost equal (49.0% men), while in South Africa, men made up 42.0% of nationally reported cases, in the rest of Africa however, males make up a larger proportion (60.0%) of nationally reported COVID-19 cases [12]. This may be due to biological differences between sexes or socio-environmental factors and norms, for instance, men might be more likely to work during the pandemic, and thus have increased exposure to the virus [13-15]. Biological differences that may account for reduced susceptibility to infection in women include estradiol induced reduced expression of angio-converting-enzyme-2 (ACE-2) [16]. In addition, women have been documented to be more likely to perceive COVID-19 as a very serious health problem, to agree with restraining public policy measures and to comply with hygiene measures such as handwashing than men [17,18]. In our study the proportion of women affected in the second wave was higher than in the first wave. This may indicate the increasing susceptibility of women in this pandemic which could be due to cultural and societal roles [15]. Previously, research has shown that women were more likely to be infected with the Ebola virus, which spread across West Africa from 2014 to 2016, as females usually serve as care providers in families and communities caring for their ill relatives in their homes and subsequently getting infected [19]. Further investigations are required to understand the gendered impacts of this pandemic. Male sex has been shown to carry a worse prognosis of COVID-19 [2,15] this was consistent with our finding of a higher case fatality among men. This could be due to biological and behavioral differences between sex such as more robust immune response in women or a higher incidence of cigarette smoking among men. Further investigations are required to understand the difference in case fatality by sex in Zambia.

More cases were reported in the second wave than in the first. This was consistent with findings from 40 other African countries that experienced or were starting to experience a second wave of cases. Among these countries, 24 (60.0%) reported a mean increase of 403% (range 10.0% - 4602.0%) in the mean number of new cases per day being reported from wave start to peak when comparing the first and the second waves [20]. This increase in numbers in the second wave could have been due to an increase in testing capacity at national level [21] and/or due to the relaxing of public health measures such as distancing and intermittent lockdowns driven by economic necessity and pandemic fatigue [20]. Additionally, mutations in the virus may lead to increased infectivity and may account for the increase in cases between waves [22]. Phylogenomic analysis of the first cases detected in Zambia with SARS-CoV-2 in March 2020 showed that it belonged to the lineage B.1.1, sharing the most common recent ancestor with viruses detected in South Africa [23]. Of 23 specimens collected during the second wave in Zambia (16^th^-23^rd^ December 2020), 22 (96.0%) of the specimens were the Beta (B.1.351) variant [24]. Due to mutations on the spike protein that increase transmissibility (N501Y) and hinder antibody binding (E484K), the Beta variant is of public health concern as it blunts naturally developed immunity and reduces vaccine efficacy [24,25]. Thus, routine phylogenomic surveillance and analysis of SARS-COV-2 specimens should form part of the outbreak response in Zambia and guide subsequent public health action such as choice of vaccine.

Worldwide, the average case fatality proportion for COVID-19 ranges from 2.0%-3.0%, in Africa it is 2.4% [20,26]. The lower overall case fatality reported in this study could be due to the lower median age of cases in Zambia as age is a well-established risk factor for severe disease and mortality [2,27]. This could be due to age-related immune changes or the higher likelihood of the presence of comorbid conditions in the elderly. The lower case fatality seen in the second wave could be due to the lower median age of cases in the second wave and the higher proportion of women affected in the second wave compared to women affected in the first wave, as mortality was significantly associated with older age and male sex in this study. Additionally, the logistical and clinical care experience gained from the first wave could have resulted in better preparation and patient care in the second wave, resulting in lower case fatality in the second wave. The number of COVID-19 cases and deaths reported in this study most likely reflects a fraction of the true toll of COVID-19 in Zambia. A population-based SARS-CoV-2 prevalence study done in Zambia showed that the number of laboratory-confirmed cases reported in official statistics underestimated SARS-CoV-2 prevalence by a factor of 92 [28] which may have been because of the high number of asymptomatic individuals reported in that study. Another study that looked at the prevalence of SARS-CoV-2 among deceased individuals at a tertiary hospital morgue in Lusaka, Zambia found a SARS-CoV-2 prevalence of 15.9% [6]. In that study, among facility deaths, only 31.6% had been tested ante mortem whilst among community deaths, none had been tested for COVID-19 ante mortem. There is a need to increase testing capacity by expanding the scope of routine COVID-19 surveillance to include asymptomatic individuals and community deaths to ascertain the true extent and spread of COVID-19 in Zambia to inform public health interventions to stop its spread.

**Limitations:** this study had several limitations. We only analyzed the positive cases and did not analyze the negative cases due to the nature of the dataset. Future analyses will include all tests conducted in Zambia, regardless of the result. Not all reported deaths were captured by this dataset; however, due to its level of completeness and large size, we were still able to identify trends that are corroborated by data from other countries, suggesting our findings might not be biased by missing data. Because this was an observational study, we cannot attribute causation between factors associated with mortality in this analysis.

**Funding:** this project has been supported by the President's Emergency Plan for AIDS Relief [PEPFAR] through the Centers for Disease Control and Prevention [CDC] under the terms of a cooperative agreement with the Zambia Ministry of Health [CoAg ID number: GH002234; CoAg name: Ministry of Health; CoAg principal investigator: Lloyd B Mulenga; CoAg funding period: 9/30/2020-9/29/2025] and the Zambia Ministry of Health.

## Conclusion

During the SARS-CoV-2 epidemic in Zambia, most deaths occurred within the community, among the elderly and among males. This indicates potential gaps in public health messaging about COVID-19. As the group most impacted by COVID-19 mortality, older persons might need enhanced outreach and linkage to care and by improving health seeking behavior. More people were infected in the second wave of COVID-19 in Zambia, and these were younger than those infected in the first wave. This highlights the need for continuously monitoring the demographic distribution of cases to guide public health intervention efforts. This can be achieved by targeted public health interventions to reduce behavioral and occupational risk factors among young adults.

### What is known about this topic

- *Worldwide males and the elderly are more at risk of SARS-COV-2 infection and of more severe clinical forms and outcomes of COVID-19 once infected;*
- *There is wave to wave variation of the demographic subset of the population affected by SARS-COV-2*.

### What this study adds

- *This study adds to the limited information on the demographic characteristics of cases and deaths in Zambia and Africa as a whole;*
- *This study establishes that most deaths due to/associated with COVID-19 in Zambia do not occur within health facilities but within communities;*
- *Even though fewer cases were recorded in the 60 and over age group most deaths occurred in this age group*.

## References

[1] Guan W, Ni Z, Hu Y, Liang W, Ou C, He J (2020). Clinical characteristics of Coronavirus Disease 2019 in China. N Engl J Med.

[2] Izcovich AI, Alberto Ragusa M, Tortosa F, Andrea Lavena Marzio M, Agnoletti C, Bengolea A (2020). Prognostic factors for severity and mortality in patients infected with COVID-19: A systematic review. PLoS One.

[3] Zambia National Public Health Institute A Centre of Excellence in Public Health Security for a Healthy Zambia.

[4] Boehmer TK, DeVies J, Caruso E, van Santen KL, Tang S, Black CL (2020). Changing Age Distribution of the COVID-19 Pandemic-United States, May-August 2020. MMWR Morb Mortal Wkly Rep.

[5] Greene DN, Jackson ML, Hillyard DR, Delgado JC, Schmidt RL (2020). Decreasing median age of COVID-19 cases in the United States-changing epidemiology or changing surveillance?. PLoS One.

[6] Mwananyanda L, Gill CJ, MacLeod W, Kwenda G, Pieciak R, Mupila Z (2020). COVID-19 deaths detected in a systematic post-mortem surveillance study in Africa. MedRxiv.

[7] Hamukale A, Hines JZ, Sinyange N, Fwoloshi S, Malambo W, Sivile S (2021). SARS-CoV-2 mortality surveillance among community deaths brought to University Teaching Hospital Mortuary in Lusaka, Zambia, 2020. MedRxiv.

[8] Chipimo PJ, Barradas DT, Kayeyi N, Zulu PM, Muzala K, Mazaba ML (2020). First 100 persons with COVID-19 Zambia, March 18-April 28, 2020. MMWR Morb Mortal Wkly Rep.

[9] Chisumpa VH, Odimegwu CO, De Wet N (2017). Adult mortality in sub-saharan Africa, Zambia: Where do adults die?. SSM Popul Health.

[10] Shaw JA, Meiring M, Flinn M, Hiemstra A, Reuter H, Stanley K (2021). Higher SARS-CoV-2 Seroprevalence in workers with lower Socioeconomic Status in Cape Town, South Africa. PLoS One.

[11] Jalali SF, Ghassemzadeh M, Mouodi S, Javanian M, Akbari Kani M, Ghadimi R (2020). Epidemiologic comparison of the first and second waves of coronavirus disease in Babol, North of Iran. Caspian J Intern Med.

[12] Globalhealth5050 (2021). The COVID-19 sex disaggregated data tracker January update report. Globalhealth5050.

[13] Patil A, Tripathy JP, Deshmukh V, Sontakke B, Tripathi SC (2020). SeXX and COVID-19: tussle between the two. Monaldi Arch Chest Dis.

[14] Wenham C, Smith J, Morgan R (2020). COVID-19: the gendered impacts of the outbreak. The Lancet.

[15] Rozenberg S, Vandromme J, Charlotte M (2020). Are we equal in adversity?. Does COVID-19 affect women and men differently? Maturitas.

[16] Liu J, Ji H, Zheng W, Wu X, Zhu JJ, Arnold AP (2010). Sex differences in renal angiotensin converting enzyme 2 (ACE2) activity are 17β-oestradiol-dependent and sex chromosome-independent. Biol Sex Differ.

[17] Johnson HD, Sholcosky D, Gabello K, Ragni R, Ogonosky N (2003). Sex differences in public restroom handwashing behavior associated with visual behavior prompts. Percept Mot Skills.

[18] Galasso V, Pons V, Profeta P, Becher M, Brouard S, Foucault M (2020). Gender differences in COVID-19 attitudes and behavior: Panel evidence from eight countries. Proc Natl Acad Sci USA.

[19] Davies SE, Bennett B (2016). A gendered human rights analysis of Ebola and Zika: locating gender in global health emergencies. International Affairs.

[20] Salyer SJ, Maeda J, Sembuche S, Kebede Y, Tshangela A, Moussif M (2021). The first and second waves of the COVID-19 pandemic in Africa: a cross-sectional study. Lancet.

[21] African Union Africa Centre for Disease Control and Prevention. AU and Africa CDC launch Partnership to Accelerate COVID-19 Testing: Trace, Test and Track.

[22] World Health Organization New COVID-19 variants fuelling Africa´s second wave.

[23] Simulundu E, Mupeta F, Chanda-Kapata P, Saasa N, Changula K, Muleya W (2021). First COVID-19 case in Zambia-Comparative phylogenomic analyses of SARS-CoV-2 detected in African countries. Int J Infect Dis.

[24] Greaney AJ, Loes AN, Crawford KH, Starr TN, Malone KD, Chu HY (2021). Comprehensive mapping of mutations to the SARS-CoV-2 receptor-binding domain that affect recognition by polyclonal human serum antibodies. Cell Host Microbe.

[25] Andreano E, Piccini G, Licastro D, Casalino L, Johnson NV, Paciello I (2020). SARS-CoV-2 escape in vitro from a highly neutralizing COVID-19 convalescent plasma. BioRxiv.

[26] Cao Y, Hiyoshi A, Montgomery S (2020). COVID-19 case-fatality rate and demographic and socioeconomic influencers: worldwide spatial regression analysis based on country-level data. BMJ Open.

[27] Hu D, Lou X, Meng N, Li Z, Teng Y, Zou Y (2021). Influence of age and gender on the epidemic of COVID-19: Evidence from 177 countries and territoriesa-an exploratory, ecological study. Wien Klin Wochenschr.

[28] Mulenga LB, Hines JZ, Fwoloshi S, Chirwa L, Siwingwa M, Yingst S (2021). Prevalence of SARS-CoV-2 in six districts in Zambia in July, 2020: a cross-sectional cluster sample survey. Lancet Glob Health.

